# Machine-learned pattern identification in olfactory subtest results

**DOI:** 10.1038/srep35688

**Published:** 2016-10-20

**Authors:** Jörn Lötsch, Thomas Hummel, Alfred Ultsch

**Affiliations:** 1 Institute of Clinical Pharmacology, Goethe - University, Theodor Stern Kai 7, 60590 Frankfurt am Main, Germany; 2 Fraunhofer Institute of Molecular Biology and Applied Ecology - Project Group Translational Medicine and Pharmacology (IME-TMP), Theodor – Stern - Kai 7, 60590 Frankfurt am Main, Germany; 3 Smell & Taste Clinic, Department of Otorhinolaryngology, TU Dresden, Fetscherstrasse 74, 01307 Dresden, Germany; 4 DataBionics Research Group, University of Marburg, Hans - Meerwein - Straße, 35032 Marburg, Germany

## Abstract

The human sense of smell is often analyzed as being composed of three main components comprising olfactory threshold, odor discrimination and the ability to identify odors. A relevant distinction of the three components and their differential changes in distinct disorders remains a research focus. The present data-driven analysis aimed at establishing a cluster structure in the pattern of olfactory subtest results. Therefore, unsupervised machine-learning was applied onto olfactory subtest results acquired in 10,714 subjects with nine different olfactory pathologies. Using the U-matrix, Emergent Self-organizing feature maps (ESOM) identified three different clusters characterized by (i) low threshold and good discrimination and identification, (ii) very high threshold associated with absent to poor discrimination and identification ability, or (iii) medium threshold, i.e., in the mid-range of possible thresholds, associated with reduced discrimination and identification ability. Specific etiologies of olfactory (dys)function were unequally represented in the clusters (p < 2.2 · 10^−16^). Patients with congenital anosmia were overrepresented in the second cluster while subjects with postinfectious olfactory dysfunction belonged frequently to the third cluster. However, the clusters provided no clear separation between etiologies. Hence, the present verification of a distinct cluster structure encourages continued scientific efforts at olfactory test pattern recognition.

Psychophysical tests assessing a subject’s olfactory performance most frequently include the assessment of a subject’s odor identification performance addressing the ability to name or associate an odor^1^,^2^. Developments of more comprehensive test batteries led to the inclusion of the assessment of a subject’s odor threshold addressing the lowest concentration of a selected odorant at which it is still perceived^3^. A third test is the assessment of a subject’s odor discrimination performance addressing the ability to distinguish different smells^4^. Contemporary olfactory test batteries include all of these components^5^ or a subset of olfactory tests^6^,^7^,^8^,^9^.

A distinct importance of the three main components of the sense of smell is an active research topic. The scientific and clinical interest dedicated this question owes to the aim at deeper understanding of the pathological mechanisms via which various different etiologies may cause olfactory dysfunction and by the desire to associate an olfactory dysfunction with a specific cause, for example in a medico-legal context. The discussion is maintained by suggestions that olfactory test measure a common source of variance^10^ that are contrasted with clinical evidence of distinct reactions of olfactory subtests to particular conditions, such as impaired odor identification but not thresholds after focal brain excision^11^ or in AIDS-related dementia^12^, or the predominant reduction of odor thresholds by drugs such as sildenafil^13^, remifentanil^14^ or tetrahydrocannabinol^15^.

Several approaches were taken to address the distinct importance of olfactory subtests reaching from correlative approaches^16^ to factor analyses^17^. In the present analysis, the problem was addressed using a data driven approach in a data set comprising olfactory thresholds, odor discrimination and odor identification scores available from 10,714 subjects. The subjects had various degrees of olfactory (dys)function associated with different underlying etiologies^18^. Unsupervised machine-learning^19^ was applied to find subgroups of patients sharing similar olfactory subtest patterns. To address the effects of common causes of olfactory deficits on the olfactory subtests, the identified patterns were subsequently assessed for their association with different etiologies of altered olfactory function.

## Methods

### Subjects and olfactory testing

The study followed the Declaration of Helsinki and was approved by the Ethics Committee of the Faculty of Medicine of the TU Dresden (number EK251112006). Informed written consent was obtained from all subjects. Subjects (age: range 6–95 years, mean ± standard deviation: 52.2 ± 17 years; sex: 6,004 men, 4,710 women) were included since they had presented at the Smell & Taste Clinic, Dept. of ORL, TU Dresden with the symptom “olfactory loss”, or they had been tested in the context of a clinical standard check or they were enrolled in research projects as healthy controls. Subjects represented several different etiologies associated with olfactory performance (Table 1).

Olfactory function was assessed using the clinically established “Sniffin’ Sticks” test battery (Burghart, Wedel, Germany^20^,^21^). As described previously^18^, the test uses felt-tip pens that contain solutions of odors. Olfactory stimulation is achieved by placing the pen with removed cap for approximately 3 s at 1–2 cm before the nostrils. **Odor thresholds** were obtained for the rose-like odor phenyl ethyl alcohol presented in 16 successive 1:2 dilution steps starting from a 4% solution using a three-alternative forced-choice task and a staircase paradigm. The odor threshold is finally estimated as the mean of the last four out of seven staircase reversals. **Odor discrimination** was determined with 16 triplets of pens, two of each triplet containing the same odor and the third a different, “target” one (for names of odors see ref. 20). **Odor identification** was determined with 16 further odors (for names of odors see ref. 20) using a four-alternative forced-choice task with presentation of a list of four descriptors for each pen.

### Data analysis

Data were analyzed using the R software package (version 3.2.3 for Linux; http://CRAN.R-project.org/22). Following statistical exploration of effects of sex^23^,^24^ and age. a sex-specific linear age correction was applied to the olfactory subtest scores. Subsequently, the probability density function (PDF) of the corrected olfactory subtest results was analyzed using the Pareto density estimation (PDE). This is a kernel density estimator particularly suitable for the discovery of groups^25^. The distribution of olfactory subtest results was analyzed by fitting a separate Gaussian mixture model (GMM) to the PDE of each subtest distribution. This was performed using our R package “AdaptGauss” (https://cran.r-project.org/web/packages/AdaptGauss/index.html) released previously^26^. Following this analysis, the probability for each subject to belong to a particular Gaussian of the obtained mixtures could be calculated as the Bayesian posterior^27^. With m = 3 Gaussians of the GMM by which the distributions of the scores of olfactory threshold, discrimination and identification were described, accommodating low, medium and high scores in each subtest, a vector of nine posterior probabilities was associated with each subject.

Unsupervised machine learning was used to identify structures within the 9 × 10,714 sized data space. Specifically, Emergent Self-organizing feature maps (ESOM) were applied in combination with the use of the U-matrix^28^. ESOM are based on a topology-preserving projection of high-dimensional data points *x*_*i*_ *ϵ R*^*D*^ onto a two dimensional self-organizing network consisting of a grid of neurons. This approach represents a topology preserving mapping of high-dimensional data points onto a two dimensional grid of neurons which was therefore favored to more common clustering algorithms such as *k*-means, Ward, complete- and average linkage^29^. The neural network consisted of a two-dimensional toroid grid of so-called neurons with 50 rows and 80 columns (n = 4,000 units). Each neuron holds a vector carrying “weights” of the same dimensions as the input dimensions, i.e., the Bayesian posteriors from GMM as described above. The weights were initially randomly drawn from the range of the data variables and subsequently adapted to the data during the learning phase that used 30 epochs respectively sweeps through the data.

A trained emergent self-organizing map (ESOM) was obtained that represented the subjects on a two-dimensional toroid map as the localizations of their respective “best matching units” (BMU), i.e., neurons on the grid that after ESOM learning carried the vector that was most similar to a subjects’ data vector. Finally, on top of this SOM grid the distance structure in the high dimensional feature space was visualized in the form of a so-called U-Matrix^29^,^30^. This was further enhanced by calculating a P-matrix that displays the point density *p*(*x*) = |{*data points x*_*i*_| *d*(*x*_*i*_*, x*) < = *r*}| estimated as the number of data points in a sphere with radius *r* around *x* at each grid point on the ESOM’s output grid. The U*-matrix combines distance structures (U-matrix) and density structures (P-matrix) into a single matrix^28^. A geographical map analogy was used to enhance the visual detection of borders of data clusters. That is, on this landscape form large heights represented large distances in the feature space while low “valleys” represented data subsets that are similar, while watersheds resembling mountain range separate data clusters. These procedures were performed using our R library “Umatrix” (M. Thrun, F. Lerch, Marburg, Germany, http://www.uni-marburg.de/fb12/datenbionik/software^31^; file http://www.uni-marburg.de/fb12/datenbionik/umatrix.tar.gz). The association of the identified clusters with the etiologies underlying a subject’s olfactory function was analyzed by means of χ^2^ statistics and by calculating the relative difference between the number of observed and the number of expected subjects in a cluster, *relDiff* = (*n*_*expected*_ – *n*_*obsered*_)/(0.5 (*n*_*expected*_ + *n*_*obsered*_)), as a metric for differentially represented items^32^. Finally, to further interpret the associations between olfactory subtests, the correlation coefficients, Spearman’s ρ^33^, were calculated for discrimination/identification, discrimination/threshold and threshold/identification, for the complete data set and separately for each etiological subset.

## Results

### Machine-learned identification of clusters of olfactory subtest results

**Figure 1:**
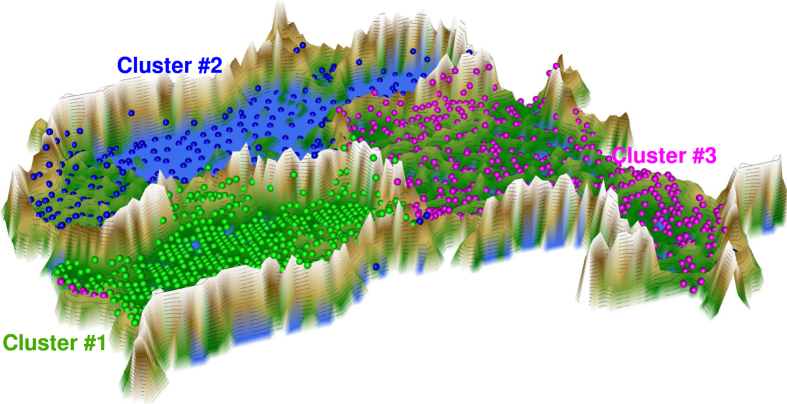
3D-display of the U-matrix representation of the olfactory subtest results pattern obtained using a projection of the data points onto a grid 4,000 neurons where opposite edges are connected (toroid grid). The U-Matrix was colored as a geographical map with brown or snow-covered heights and green valleys. The cluster structure emerges from visualization of the distances between neurons in the high-dimensional space by means of a U-Matrix. Thus, on the 3D-display the valleys indicate subjects sharing similar subtest patterns while the mountain ranges with “snow-covered” heights separate the clusters (for a top view, see Fig. 3 left). The dots indicate the so-called “best matching units” (BMUs) of the self-organizing map (SOM), which are those neurons whose weight vector after SOM training was most similar to the input vector carried by particular subjects. The BMU coloring corresponds to the cluster membership. The figure displaying a geographical map analogy has been created using our R library “Umatrix” (M. Thrun, F. Lerch, Marburg, Germany, http://www.uni-marburg.de/fb12/datenbionik/software ; file http://www.uni-marburg.de/fb12/datenbionik/umatrix.tar.gz).

Following projection of the vector space comprising the olfactory subtest results of each subject onto a toroid grid of 50 × 80 = 4,000 neurons and training of a self-organizing map, a U*-matrix was displayed on top of this SOM. This provided an emergent self-organizing feature map (ESOM) in which large U-heights in the U*-matrix indicated a large gap in the data space whereas low U-heights indicated that the points are close to each other in the data space indicating a distance based cluster structure in the data set. On a 3D-display of this U-matrix (Fig. 1), valleys, ridges and basins demonstrate how the distance based cluster structure could be visualized in the data set.

**Figure 2:**
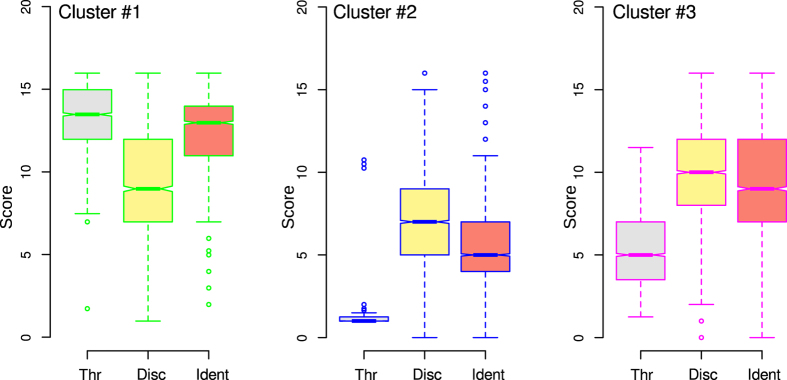
Cluster–specific (Fig. 1) olfactory subtest results (Thr = olfactory threshold, Disc = odor discrimination, Ident = odor identification). The box borders and lines colors are set at the coloring of the clusters in Fig. 1. The minimum, quartiles, median (solid horizontal line within the box), and maximum are used to construct a “box and whisker plot. The notches display a confidence interval around the median. The figure has been created using the R software package (version 3.2.3 for Linux; http://CRAN.R-project.org/22).

This cluster structure seen on the U*-matrix provided three main clusters (green, blue and magenta points in Fig. 1). The clusters were characterized (Fig. 2) by (cluster #1) a low olfactory threshold (median at dilution step #13.5) associated with good odor discrimination and identification performance, or by (cluster #2) a very high olfactory threshold (median at the first dilution step) that was associated with absent to poor odor discrimination and identification ability, or by (cluster #3) a medium olfactory threshold (median at dilution step #5). i.e., at the lower part of the mid-range of possible thresholds according to the test battery and associated with still preserved odor discrimination and identification ability.

### Relationship of olfactory subtest patterns and etiologies associated with olfactory function

**Figure 3:**
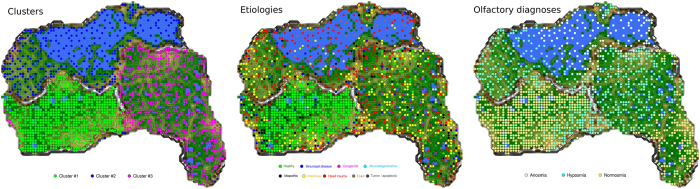
U-matrix representations of the olfactory subtest results pattern obtained using a projection of the data points onto a toroid grid 4,000 neurons where opposite edges are connected. The U-Matrix was colored as a geographical map with brown or snow-covered heights and green valleys. Thus, valleys indicate clusters and watersheds indicate borderlines between different clusters. The dots indicate the so-called “best matching units” (BMUs) of the self-organizing map (SOM), which are those neurons whose weight vector is most similar to the input. A single neuron can be the BMU for more than one data pint or subject, hence, the number of BMUs may not be equal to the number of subjects as in the present case. The BMUs were differently colored to analyze the distribution of subjects across the cluster structure of the data space. Left: Localization of cluster memberships on the U-matrix, for comparison with the further presentations, using a coloring for the BMUs corresponding to the membership to the machine-learned clusters of olfactory subtest results patterns. Middle: Distribution of etiologies associated with the subjects’ olfactory function across the U-matrix respectively cluster structure. Right: Distribution of the olfactory diagnoses (anosmia, hyposmia, normosmia) across the U-matrix respectively cluster structure. The figures displaying geographical map analogies have been created using our R library “Umatrix” (M. Thrun, F. Lerch, Marburg, Germany, http://www.uni-marburg.de/fb12/datenbionik/software ; file http://www.uni-marburg.de/fb12/datenbionik/umatrix.tar.gz).

**Figure 4:**
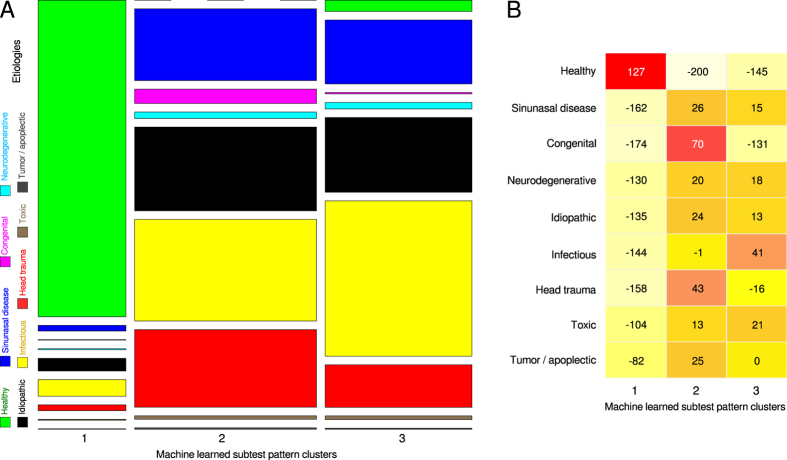
Summary plot of the associations of clusters with etiologies of olfactory (dys)function. (**A**) Mosaic plot representing a contingency table of the etiologies suspected to be associated with the subjects’ olfactory function versus the machine-learned clusters of olfactory subtest results. The size of the cells as proportional to the number of subjects included; the color code is similar to that used in Fig. 3 middle. (**B**) The same contingency table was assessed for overrepresentation of etiologies among machine-learned clusters, which was analyzed using the relative differences of observed and expected observations^32^. In the matrix plot, the cells are equally sized and display the relative differences; the color code ranges from light yellow indicating underrepresentation to red indicating overrepresentation. The figure has been created using the R software package (version 3.2.3 for Linux; http://CRAN.R-project.org/22). Specifically, the plots were been drawn using the using the “mosaicplot” (left) or “heatmap.2” (right) functions, the latter being part of the R package “gplots” (Warnes G. R.; https://cran.r-project.org/web/packages/gplots/index.html); with the build-in clustering of the plotting routine disabled (R switches “Colv = FALSE, Rowv = FALSE”).

**Figure 5:**
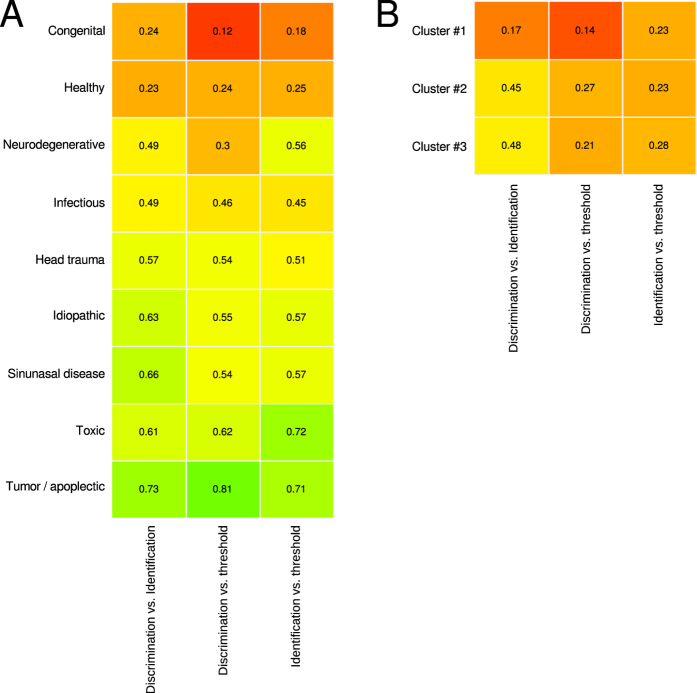
Matrix plots of the correlations among the olfactory subtest results (discrimination/identification, discrimination/threshold, threshold/identification). The numbers in each cell indicate the Spearman correlation coefficients ρ^33^. The cells are color coded according to the squared correlation coefficients, with red indicating weak correlation (p < 0.2^34^), yellow indicating medium and green indicating stronger correlation’s (p > 0.7), with smoothed transitions between the colors. Panel A shows the correlations separately for the different etiologies of olfactory dysfunction, and panel B shows the same for the EXOM/U-matrix based clusters. The figure has been created using the R software package (version 3.2.3 for Linux; http://CRAN.R-project.org/22). Specifically, the plot has been drawn using the using the “heatmap.2” function of the R package “gplots” (Warnes G. R.; https://cran.r-project.org/web/packages/gplots/index.html). The build-in clustering of the plotting routine has been disabled (R switches “Colv = FALSE, Rowv = FALSE”) and the etiologies have been ordered for increasing average correlation coefficients.

A projection of the etiologies noted during clinical subject enrolment onto the cluster structure obtained on the ESOM/U*-matrix discouraged a strong meaningful association. Specifically, while a clear overrepresentation of healthy subjects in the cluster with consistently good olfactory subtest results (cluster #1) was observed, the pathological etiologies seemed to be scattered across the other clusters (Fig. 3 middle). However, despite this impression the association of specific etiologies of olfactory (dys)function to the machine-leaned clusters was unequal at a high statistical significance level (χ^2^ = 8916.6, df = 16, p < 2.2 · 10^−16^) that remained significant when excluding the healthy subjects (χ^2^ = 396.26, df = 14, p < 2.2 · 10^−16^). Exploration of the representation of the etiologies among the clusters (Fig. 4) indicated (i) a clear overrepresentation of healthy subjects in cluster #1 where all pathological etiologies seemed to be underrepresented, (ii) an overrepresentation of subjects with congenital anosmia and possibly of those with head trauma in cluster #2 where healthy subjects were clearly underrepresented and (iii) an overrepresentation of subjects postinfectious olfactory dysfunction in cluster #3. However, consistent with the distribution of the subjects across the clusters on the ESOM/U-matrix, the clusters provided no clear separation between etiologies associated with the subjects’ olfactory function. The distribution of 3,326 anosmic patients, 4,664 hyposmic patients and 2,624 patients or healthy subjects with normosmia diagnosed according to the criteria of the “Sniffin’ Sticks” test battery was consistent with a known broad variability of the functional consequences of most etiologies associated with normal or altered olfactory function (Fig. 3 right). Finally, the correlations between olfactory subjects were statistically significant at the global level (discrimination/identification: ρ = 0.551, discrimination/threshold: ρ = 0.464, threshold/identification: ρ = 0.71, all p < 10^−5^). The correlations between olfactory subtest results also varied among the different etiologies (Fig. 5) from virtually absent (ρ < 0.2) to strong (ρ > 0.7)^34^, and the same analyses applied to the ESOM/U-matrix based clusters produced similar results (Fig. 5).

## Discussion

Results of this analysis support the existence of structure among olfactory subtest results. This was obtained using a data-driven approach that did not predict such structure. In the unsupervised machine-learning approach, the goal was to discover structure, i.e., the first step of the present analysis was not to analyze implications of an observed or assumed cluster structure, but to establish whether or not such a structure exists in the pattern of the results of the three olfactory subtests. Only subsequently to establishing a cluster structure, the analysis aimed at associating different etiologies of possible olfactory dysfunction with the clusters identified in the data space. This differs from previous hypothesis-driven approaches to a differential importance of olfactory subtest results that used a correlational approach to test, whether the different subtests would correlate with different cognitive functions^17^ or analyzed similarities between subtest results without aiming at finding subgroups of patients sharing similar olfactory test pattern^17^. Interestingly, in one of these previous studies in 97 subjects no major difference was found between nine different tests of various aspects of olfactory function (tests of odor identification, discrimination, detection, memory, and suprathreshold intensity and pleasantness perception)^10^, although the number of subjects might have been too small to detect subtle differences considering the large variance typically seen with these olfactory tests.

With the observation that the different etiologies associated with different outcomes of the olfactory test battery were unevenly distributed across the clusters of olfactory subtest results, the present analysis is in line with previous reports about a different vulnerability of single subtest results to the effect of various causes of olfactory dysfunction (e.g. refs 12,35 and 36). Moreover, the different degrees of subtest correlation (Fig. 5) between the different etiologies, or clusters, also hint at distinct affections of olfactory function by diseases causing its deterioration. However, while previous reports often drew an unrestricted positive conclusion about the finding of such differences, the present results include the restriction that the observed pattern provide no clear picture of the distinct influence of etiologies of olfactory dysfunction on olfactory subtest pattern. The clusters could be only partially associated with an overrepresentation of a few etiologies, however, other etiologies seemed to produce often the same pattern. This could have been an effect of poor clustering. However, this is contrasted by previous demonstrations that the chosen ESOM/U-matrix method is able to identify clusters in data where classical clustering methods fail as on data of the Fundamental Clustering Problems Suite (FCPS) published as benchmark problems for clustering algorithms^37^. Popular clustering algorithms such as k-means, Ward, complete- and average linkage fail to cluster this data set correctly because these algorithms imply a geometrical model of the cluster structure. That is, *k*-means clustering implies a spherical shape of the clusters, while Ward hierarchical clustering implies a hyperelliptic shape. If this implicit assumption about cluster shape does not fit the cluster structure in the data, the clustering will fail. By contrast, Emergent Self-organizing feature maps (ESOM) using the U-matrix represent a topology preserving mapping of high-dimensional data points onto a two dimensional grid of neurons. Thus, the observed only weak association of etiologies with a distinct pattern of olfactory subtest results is probably a true observation. This suggests that for a complete distinctive pattern, further olfactory test results are needed in addition to the three subtests.

The most pronounced association of a particular cluster of olfactory subtest result pattern and an etiology underlying olfactory dysfunction was observed, besides for healthy subjects and cluster #1, for subjects suffering from congenital anosmia and, to a lower extent, for subjects with postinfectious olfactory dysfunction. Patients assigned to a congenital lack of olfaction were those who could not recall any perception of odors throughout their lives due to an agenesis/hypogenesis of their olfactory bulbs. They are typically diagnosed with “congenital anosmia” at an age of approximately 14–16 years; parents often (wrongly) believe that they are ill-observing and irresponsible parents. People with congenital anosmia seem to have a normal life^38^. Subjects with postinfectious olfactory dysfunction are typically women older than 50 years. The infection seems to damage the olfactory epithelium which causes olfactory loss. that is frequently incomplete, with a good chance for recovery (seen in about 2/3 of the patients)^39^.

Results encourage further pursuing pattern of olfactory function typical for certain etiologies. While three subtests probably are not enough, olfactory assessments include more options and therefore, the expectation that disease-typical olfactory pattern can be identified seems reasonable. It is in line with the increasing recognition of structure in human olfactory data^18^. This is likely to be of diagnostic utility. A clear pattern of olfactory test results associated with a particular etiology would also be desirable in medico-legal settings where, for example, a compensation for a claimed incident with lost olfactory function could be disputed on a basis that the pattern of olfactory test results correspond to a postinfectious dysfunction making a claimed traumatic or toxic etiology unlikely. Moreover, while present results suggest a differential modulation of olfactory subtest results by the various causes of dysfunction as reflected in the significant differences and apparent differences in the correlation levels of the three subtest among different etiologies or clusters, the results also clearly indicate that olfactory subtest results alone provide no sufficient information for a sensitive and specific association with a particular pathophysiological cause of the olfactory dysfunction. Possibly, among additional data needed to successfully approach the distinct olfactory pathologies is molecular information about the mechanisms via which different causes such as viral infection, toxic exposure, tumors or trauma influence the sense of smell at several different level exceeding the sensory perception of odors that was in the focus of the present analysis.

## Conclusions

Using a data-driven approach, a cluster structure was found by applying unsupervised machine-learning^19^ to the results of olfactory subtests addressing olfactory thresholds, odor discrimination and odor identification. These are the olfactory dimensions implemented most frequently in the clinical testing of the human sense of smell. The observed pattern differed statistically significantly among various etiologies of olfactory dysfunction. While the three subtest results seem still insufficient to provide a distinction of the various different etiologies that can lead to olfactory dysfunction, the verification of a distinct cluster structure in the pattern of olfactory test results clearly encourages continued scientific efforts at olfactory test pattern recognition. Adding additional components of olfactory diagnostics there is a reasonable chance to succeed in a classification of olfactory dysfunction for the underlying etiology.

## Additional Information

**How to cite this article**: Lötsch, J. *et al.* Machine-learned pattern identification in olfactory subtest results. *Sci. Rep.* **6**, 35688; doi: 10.1038/srep35688 (2016).

## Figures and Tables

**Table 1:** Demographics of the enrolled subjects, separately for the subjects’ sex and the physiological or pathological (etiology) condition associated with the subjects’ olfactory functional acuity.

Etiology	Men		Women	
	n (% within etiology)	Age (mean, SD, range) [years]	n (%)	Age (mean, SD, range) [years]
Healthy	928 (44.2)	35.9 ± 16.4, 6–90	1171 (55.8)	35 ± 16.2, 7–90
Sinunasal disease	852 (52)	56.2 ± 12.7, 18–87	785 (48)	54.4 ± 12.8, 14–86
Congenital	79 (38.9)	30.8 ± 16.3, 8–65	124 (61.1)	28.9 ± 16.4, 7–74
Neurodegenerative	91 (57.6)	64.4 ± 10.8, 28–83	67 (42.4)	64.7 ± 11.3, 37–89
Idiopathic	928 (47.6)	61.4 ± 13.6, 11–89	1021 (52.4)	58.4 ± 13.9, 15–90
Infectious	961 (31.5)	58.8 ± 12.6, 11–86	2092 (68.5)	59.8 ± 11.2, 12–95
Head trauma	801 (53.7)	46.9 ± 15, 9–83	690 (46.3)	51.1 ± 15.1, 11–82
Toxic	52 (57.8)	60.8 ± 12.4, 19–76	38 (42.2)	59.6 ± 14.6, 14–80
Tumor/apoplectic	18 (52.9)	61.1 ± 13.1, 25–79	16 (47.1)	58 ± 14.7, 28–88

SD: standard deviation.
